# World AIDS Day 2007: AIDS at 26, are we there yet?

**DOI:** 10.1186/1742-4690-4-86

**Published:** 2007-12-01

**Authors:** Kuan-Teh Jeang

**Affiliations:** 1 The National Institutes of Health, Bethesda, MD, USA

## Abstract

This editorial comments on selected progress made in combating the acquired immune deficiency syndrome (AIDS) after 26 years and some of the remaining challenges.

## 

It has been 26 years since the acquired immune deficiency syndrome (AIDS) was first recognized (see review [1]). In the ensuing time, AIDS has become an unprecedented global pandemic. Today, approximately 33 million people worldwide are infected with HIV, the virus that causes AIDS. In 2007, 2.5 million people became newly infected; and around 2.1 million died of AIDS in 2006. Each day, ~10,000 new individuals become HIV seropositive with 95% residing in resource-poor developing nations. A cumulative global count shows that more than 25 million people have already died from AIDS, a number exceeding 60 times the total American casualties in World War II. Regrettably, half of all people are infected with HIV before age 25, and are killed by AIDS before they turn 35.

## Progress and Challenges

More than a quarter century of AIDS later, where do we stand against this disease? A couple of advances amongst many warrant measured optimism. First, we have made remarkable strides in developing antiviral drugs or antiretrovirals (ARVs). Since 1996, ARVs have saved an estimated 3 million life-years in the United States alone. An upside to this therapeutic advance is that currently more than 2 million HIV-positive people are being treated with ARVs. While drug resistant viruses continue to be a significant issue [2], this past year saw the emergence of a new class of drugs targeted against the HIV-1 integrase enzyme [3]. Different from inhibitors that target the reverse transcriptase and protease enzymes and drugs that affect viral entry into the cell, this new integrase inhibitor will prevent the viral DNA from inserting itself into the host cell genome. A downside to treatment remains that still less than 20% of the world's population has access to HIV drugs and prevention programs, and that for every one person who gains therapy, six others become newly HIV-infected without the prospect of future treatment. Ongoing investments from the United States President's Emergency Plan for AIDS Relief [4], the Global Fund to Fight AIDS, Tuberculosis and Malaria, and many other programs are making steady progress in attempting to turn the tide on worldwide treatment access. Second, a welcome development is the recent documentation that circumcision reduces by approximately 50% a man's risk of acquiring AIDS sexually. This piece of good news suggests that there is still much to be gained through public health prevention measures. Independent of drug therapy, education, condoms, circumcision, abstinence, and other intervention strategies may yield significant and yet unharvested global benefits.

There have also been two notable recent disappointments. The first is the serious setback of the failed clinical vaccine trial from the collaborative efforts of Merck & Co., Inc., the US National Institute of Allergy and Infectious Diseases (NIAID), and the HIV Vaccine Trials Network (HVTN). In this sizable clinical trial, 49 of 914 vaccinated men tested positive for HIV, compared to 33 of 922 men who received a placebo vaccine [5]. These results illustrate a disappointing lack of vaccine efficacy and dash the hope that a useful HIV-vaccine will be available any time soon. As the Merck vaccine was based on the induction of cellular immunity by HIV proteins expressed from an adenoviral vector, we will likely see a return to attempts to modify the viral envelope protein in such a way to induce neutralizing antibodies. This seems a formidable task that requires innovative approaches, as all the standard ways to make such an envelope immunogen have failed. A second less visible but perhaps equally troubling concern is our continued inability to develop a safe and effective anti-HIV microbicide for women [6]. In sub-Saharan Africa, women between the ages of 15–24 are three times more likely than men to become infected with HIV. Our failure to empower women to protect them against HIV/AIDS poses a sobering challenge.

## Leadership

The theme of this 20^th ^World AIDS Day is "leadership". This is a day to consider how we can exercise leadership on new innovations and vision. With increased globalization, today's world is much different from what it was 26 years ago; and very likely, the world will be further different in 26 more years. Several respected sources have projected that by 2040 China will have overtaken the United States to become the world's largest economy with India capturing third place. Official AIDS statistics in 2005 place China's HIV-cases at 650,000. With her population of 1.3 billion people, China's future HIV numbers will surely rise [7]. Nevertheless, the good news is that China's economy is robust and has amassed an estimated foreign reserve of over 1.4 trillion US dollars. In the near future, one could expect China to begin contributing to economic leadership in the global fight against AIDS. Perhaps 2008, the year of the 29th Olympic games to be held in Beijing, would present an excellent time for China's initiative in the service of global human health.

Today is also a day for the AIDS/HIV medical and research communities to reflect on new ideas, new targets [8] and to call on fresh voices. One senior researcher commented recently that he has been going to major HIV meetings for the last fifteen years and have heard over that period largely the same voices speak about AIDS vaccines. On World AIDS Day 2007, let's mark the progress already accomplished, persevere in good research while seeking new approaches from unheard voices. AIDS at 26, are we there yet? Not quite.
